# Potentially avoidable inter-facilit transfer from Veterans Health Administration emergency departments: A cohort study

**DOI:** 10.1186/s12913-020-4956-6

**Published:** 2020-02-12

**Authors:** Nicholas M. Mohr, Chaorong Wu, Michael J. Ward, Candace D. McNaughton, Kelly Richardson, Peter J. Kaboli

**Affiliations:** 1 Center for Comprehensive Access Delivery Research & Evaluation (CADRE), VA Iowa City Healthcare System, Iowa City, IA USA; 2 Department of Emergency Medicine, University of Iowa Carver College of Medicine, Iowa City, USA; 3 Department of Anesthesia, University of Iowa Carver College of Medicine, 200 Hawkins Drive, 1008 RCP, Iowa City, IA 52242 USA; 4 Institute for Clinical and Translational Sciences, University of Iowa, Iowa City, Iowa USA; 5 Tennessee Valley Healthcare System VA Medical Center, Nashville, Tennessee USA; 6 Department of Emergency Medicine, Vanderbilt University Medical Center, Nashville, USA; 7 Department of Internal Medicine, University of Iowa Carver College of Medicine, Iowa City, Iowa USA

**Keywords:** Emergency service, hospital, Regionalization, Rural health services, Hospitals, rural, Veterans health

## Abstract

**Background:**

Inter-facility transfer is an important strategy for improving access to specialized health services, but transfers are complicated by over-triage, under-triage, travel burdens, and costs. The purpose of this study is to describe ED-based inter-facility transfer practices within the Veterans Health Administration (VHA) and to estimate the proportion of potentially avoidable transfers.

**Methods:**

This observational cohort study included all patients treated in VHA EDs between 2012 and 2014 who were transferred to another VHA hospital. Potentially avoidable transfers were defined as patients who were either discharged from the receiving ED or admitted to the receiving hospital for ≤1 day without having an invasive procedure performed. We conducted facility- and diagnosis-level analyses to identify subgroups of patients for whom potentially avoidable transfers had increased prevalence.

**Results:**

Of 6,173,189 ED visits during the 3-year study period, 18,852 (0.3%) were transferred from one VHA ED to another VHA facility. Rural residents were transferred three times as often as urban residents (0.6% vs. 0.2%, *p* < 0.001), and 22.8% of all VHA-to-VHA transfers were potentially avoidable transfers. The 3 disease categories most commonly associated with inter-facility transfer were mental health (34%), cardiac (12%), and digestive diagnoses (9%).

**Conclusions:**

VHA inter-facility transfer is commonly performed for mental health and cardiac evaluation, particularly for patients in rural settings. The proportion that are potentially avoidable is small. Future work should focus on improving capabilities to provide specialty evaluation locally for these conditions, possibly using telehealth solutions.

## Background

Over 2.4 million Veterans seek care from Veterans Health Administration (VHA) emergency departments (EDs) each year, and 40% of these Veterans live in rural America [1]. Providing high quality emergency care in low-volume centers is challenging, and several prior studies have suggested that clinical outcomes are worse in low-volume rural hospitals [2–9]. These volume-outcome relationships could be attributable to provider training and experience, staffing, or resource allocation in low-volume facilities [10].

In many low-volume EDs, inter-facility transfer is used as a strategy for moving patients rapidly to hospitals equipped to care for them [11, 12]. Well-defined transfer networks have been developed for trauma and stroke care, but many patients with other conditions are transferred from EDs [11–14]. Both over-triage (transferring patients unlikely to benefit) and under-triage (failing to transfer patients likely to benefit) have been reported, and for some conditions the rate of potentially avoidable transfer (PAT) is high [15–18]. While 1.5% of US ED patients are transferred, that proportion can be much higher in rural hospitals, and access to specialists, technology, available inpatient capacity, and organizational factors can drive transfer practices [11].

The VHA provides critical emergency and specialty care to rural Veterans. All Veterans who present for care are evaluated and have standard diagnostic tests performed, but some that present to smaller facilities require transfer to larger facilities for diagnostic or therapeutic procedures or for availability of inpatient services. While inter-facility transfer provides access to care that would otherwise be unavailable in rural communities, it also imposes a substantial hardship for rural Veterans and their families by displacing them and increasing costs of care. Inter-facility emergency transfers often occur when Veterans are most vulnerable due to their acute illness, and complex care coordination can contribute to delays and triage mismatch [12, 19].

The objective of this study was to describe ED-based transfer patterns within in the VHA system, with a focus on potentially avoidable transfers (PAT). This analysis is the first step in developing targeted interventions such as ED-based telehealth to decrease the number of avoidable transfers and improve efficiency of inter-facility transfer within the VHA. To accomplish that objective, our goals were to (1) describe the VHA-to-VHA transfer population, (2) identify patient and health system factors associated with PAT, and (3) define geographic “hot spots” of high volumes of potentially avoidable transfer as a first step toward developing regional interventions. Our hypothesis was that geographic and diagnostic categories exist that are related to PATs, and that these transfers would disproportionately rural Veterans in smaller EDs on nights and weekends.

## Methods

### Study design and setting

This cohort study included all Veterans treated in VHA EDs and transferred to another VHA hospital between January 2012 and December 2014. This timeframe was selected to include only cases before implementation of *International Classification of Diseases, 10th edition* (ICD-10) in 2015. Data were abstracted from the VHA Clinical Data Warehouse (CDW), which contains national data from clinical and administrative data systems collating patient-level, visit-level, provider-level, and institution-level information. This project was determined not to be human subjects research by the local Institutional Review Board (quality improvement), and this study is reported in accordance with the STrengthening the Reporting of OBservational Studies in Epidemiology (STROBE) statement [20].

### Selection of participants

All adults who presented to one of the 120 VHA EDs were included in this study if they were transferred to another VHA acute care hospital. Patients presenting to urgent care clinics were excluded, and patients who were discharged to home, admitted locally, transferred to a non-VHA hospital, or who died in the ED were also excluded. Patients presenting by ambulance and diverted to a non-VHA facility were not included in this cohort. While some selection likely occurs in where Veterans receive emergency care, only patients who were treated in VHA EDs were included. Transferred patients were identified by linking the cohort of ED visits with all inpatient and ED visits (regardless of hospital) within 24 h after index ED arrival. Any patient who (1) had an inpatient or ED visit within 24 h at another VHA facility and (2) did not have a local hospital admission was considered to have been transferred to a VHA hospital. The 24-h visit window was permitted to allow for transfers that crossed midnight and therefore occurred on different dates.

### Facility-level variables

Institutional variables were linked from the triennial VHA ED and Urgent Care Medical Director survey (collected in 2017) administered by the VHA Healthcare Analysis and Information Group (HAIG). This HAIG survey is administered to every medical director, with one response per facility (100% response rate). ED visits were identified by inclusion in the Outpatient Data File, limited to Clinic Stop Code of 130 (emergency departments).

### Definitions

For the purposes of this study, *index hospital* indicates the hospital of the first ED visit, and *referral hospital* indicates the destination hospital after inter-facility transfer. An *ED transfer* is a transfer with a referral hospital destination of an ED, and an *inpatient transfer* is a transfer with a destination of an inpatient service (either inpatient status or observation status) without an ED visit at the referral hospital. For the purposes of this study, inpatient and observation status were considered equivalent. *Potentially avoidable transfers* (PAT) were defined as transfers in which the patient was either discharged from the referral hospital ED or the patient was admitted to the referral hospital for less than 24 h, discharged alive, and no procedures were coded. This definition is consistent with previous work in this area [17] and was intended to identify patients who were discharged quickly without a procedure that might have required specialty care. Specialist consultations were not considered to be procedures for the definition of PAT, because non-procedural opinions in many cases could be rendered without transfer. This definition was targeted to identify patients whose transfer may have been avoidable if real-time specialty telemedicine were available at index hospitals. The definition was not intended to suggest that PATs were inappropriate or could have been avoided with current resources. *Non-VHA transfers* were defined as having ED or inpatient visits reimbursed by the VHA to non-VHA hospitals within 24 h of the index ED visit (using VHA fee basis files), which occurs when VHA pays for care rendered for VHA-covered Veterans.

*Primary discharge diagnosis* was categorized based on the Clinical Classification Software (CCS) developed by the Agency for Healthcare Research and Quality (AHRQ) Health Care Utilization Project (HCUP), which groups primary ICD-9 diagnosis codes into mutually exclusive categories in a multi-level tiered system [21]. *Procedures* were defined by the HCUP Surgery Flag Software, which identifies invasive surgical procedures from Current Procedural Terminology (CPT) and ICD-9 procedure codes. For the purposes of this project, we used the *broad definition* of surgical procedures, which includes diagnostic procedures like cardiac catheterization and fiberoptic endoscopy if they are invasive, despite no therapy being performed. *Rural* Veterans were defined according to the address of residence, and classified according to Rural-Urban Commuting Areas (2 category approximation) [22]. *Regional variation* within the VHA was reported within Veteran Integrated Service Network (VISNs), a geographic classification whereby VHA facilities are organized into one of 21 regions. A *follow-up visit* within index or referral facilities was defined as an outpatient visit to any non-ED clinic within 45 days of hospital discharge to either the index hospital or referral hospital, respectively. All variables used in the analysis are included in Additional file 1.

### Estimate of transfer distances

The driving distance between pairs of VHA hospitals for each transfer was calculated in miles. All geographic analysis and mapping was performed using ArcGIS v.10.6 (Environmental Systems Research Institute, Redlands, California).

### Outcomes

The primary objective of this study was to identify common diagnoses, geographic regions, and health system factors associated PAT (primary outcome). Diagnoses were categorized into diagnosis group, and subcategories were examined to identify specific groups at highest risk of PAT. Secondary analyses included a temporal analysis of transfer patterns (e.g., day of the week, time of day), a description of the distance of transfer, and an estimate of the association between inter-facility transfer, follow-up visits, and mortality. Mortality was defined by using the date of death in the Veterans beneficiary record, it is was defined as death within 30 days.

### Analysis

Inter-facility transfers were identified and then classified as potentially avoidable or not avoidable. Descriptive summary statistics describe the population of transferred patients overall and stratified by diagnosis, rurality, procedures performed after transfer, and geography. The unit of analysis was the ED visit.

Factors associated with PAT were identified using bivariate analysis (e.g., chi-squared for dichotomous predictors and t-test or Wilcoxon rank-sum test for continuous predictors, as appropriate). Because of the very large sample size, the investigators purposely selected factors based on a priori-defined hypotheses and where differences between the groups were clinically relevant, since statistical testing was able to identify very small and clinically irrelevant differences. Variables associated with PAT in bivariate analyses were included in an explanatory multivariable logistic regression model to identify the independent contribution of each of the constituent variables. All statistical tests are reported as two-tailed tests and were considered significant if *p* < 0.05. All analyses were performed with SAS 9.4 (SAS Institute, Cary, NC). The sponsor did not contribute to the analytic plan or the reporting in any way.

## Results

**Fig. 1:**
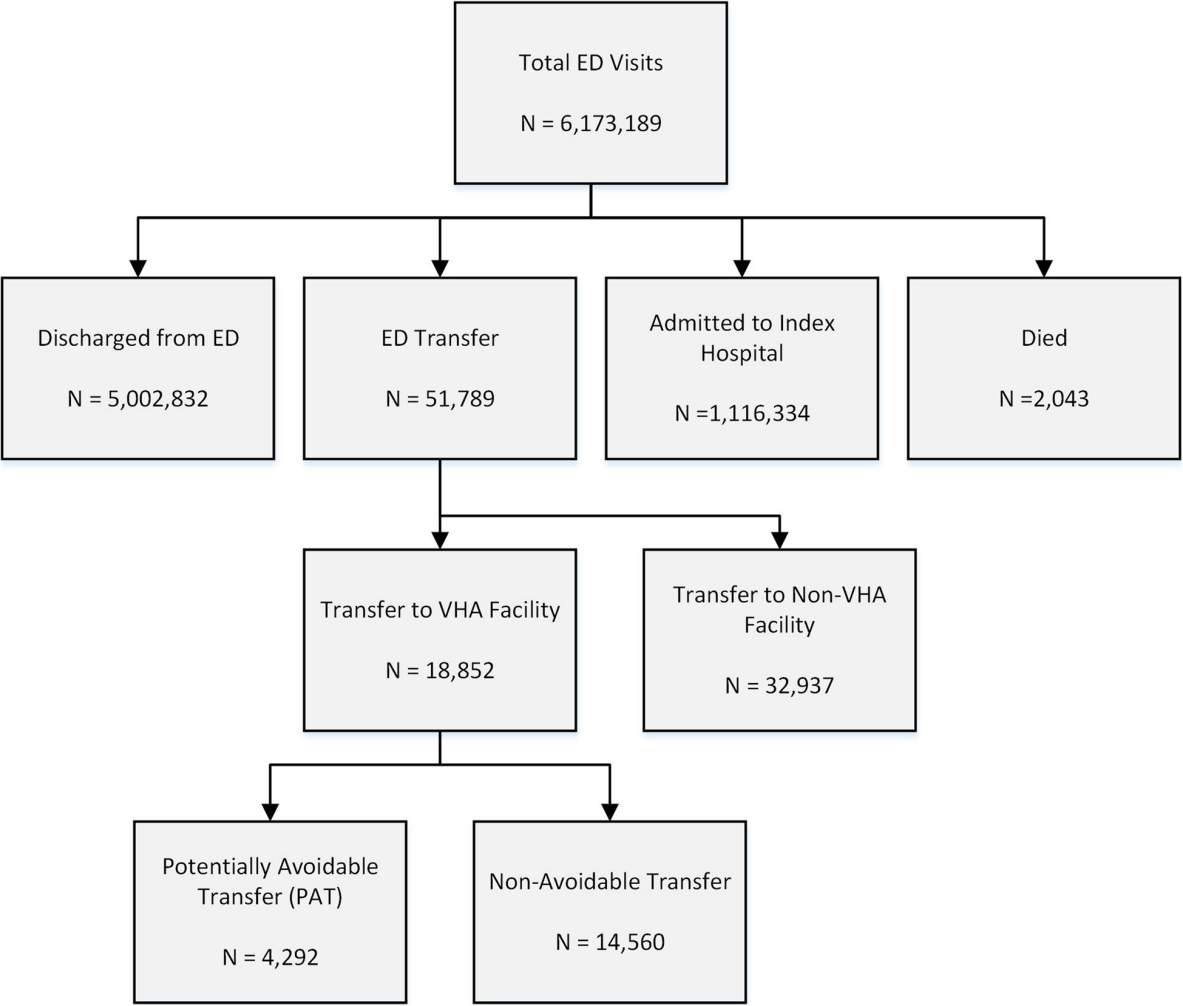
Flow diagram of study participants

Over the 3-year study period, there were 6,173,189 VHA ED visits, of which 18,852 (0.3%) were transferred to another VHA hospital (Fig. 1). The mean age of patients treated in the ED was 59 years, and 90% were male. VHA EDs treated a median of 15,989 patients annually (interquartile range 9895-22,341). Eighteen percent of the total cohort was admitted to the index VHA hospital. Rural Veterans (classified by home address) were at higher risk of VHA inter-facility transfer than urban Veterans (0.6% vs. 0.2%, *p* < 0.001). Of the total patients transferred from a VHA ED, 36% were transferred to another VHA facility.

Of VHA transfers, 46% (*n* = 8639) were transferred to another VHA ED, and the rest were transferred to another VHA facility inpatient unit. Median transfer distance was 81.5 miles (IQR 35.6–128.1 miles). PATs were identified in 22.8% (*n* = 4292) of all VHA inter-facility transfers. Of the total population with PATs, 74.6% (*n* = 3355) were discharged from the referral ED, while the rest were discharged after a brief inpatient or observation stay. While 30-day mortality of transferred patients was low overall after VHA transfer (*n* = 487, 2.6%), 65 patients died after being discharged with PAT (1.5%).

Factors associated with PAT include transfer to the referral ED (instead of inpatient unit), diagnosis, and location of transfer (Table 1). Although transfer overall was more common for rural Veterans, PAT was less prevalent (20.8% for rural Veterans vs. 23.9% for urban Veterans, *p* < 0.001). PAT was more common during nights, evenings, and weekends compared with weekday transfers (23.7% vs. 19.8%, p < 0.001), and the association between off-hours transfers and PAT was stronger for rural transfers than for urban transfers (*p* = 0.008).

**Table 1:** Patient and hospital-level factors associated with VHA-to-VHA ED inter-facility transfer, 2012–2014

	Non-Transfer (*n* = 6,154,146)	Non-Avoidable Transfer (*n* = 14,560)	Potentially Avoidable Transfer (*n* = 4292)
Age, y (SD)	58.8 (16.0)	58.9 (15.1)	56.8 (15.5)
Male, n (%)	5,512,967 (90)	13,624 (94)	3930 (92)
Rurality of Residence			
Urban, n (%)	5,078,808 (89)	9374 (75)	2899 (78)
Large Rural, n (%)	343,642 (6)	1678 (13)	420 (11)
Small Rural, n (%)	160,163 (3)	826 (7)	226 (6)
Isolated Rural, n (%)	135,530 (2)	653 (5)	177 (5)
Day of the Week			
Monday, n (%)	1,050,212 (17)	2461 (17)	706 (17)
Tuesday, n (%)	978,139 (16)	2260 (16)	642 (15)
Wednesday, n (%)	934,969 (15)	2157 (15)	658 (15)
Thursday, n (%)	913,478 (15)	2131 (15)	682 (16)
Friday, n (%)	920,805 (15)	2171 (15)	552 (13)
Saturday, n (%)	679,584 (11)	1637 (11)	490 (11)
Sunday, n (%)	635,672 (10)	1743 (12)	562 (13)
Time of Day			
8a-5p Mon-Fri, n (%)	3,199,845 (52)	6998 (48)	1720 (40)
Evenings, nights, and weekends, n (%)	2,913,014 (48)	7562 (52)	2572 (60)
Transfer Location			
ED, n (%)	N/A	5427 (37)	3212 (75)
Inpatient, n (%)	N/A	9133 (63)	1080 (25)
Hospitalization, n (%)	1,083,322 (18)	14,533 (99)	1435 (33)
Hospital Length of Stay, d (median, IQR)	4 (2, 7)	5 (3, 9)	1 (1, 1)
Number of ED beds	15.8 (10.8)	11.8 (7.3)	12.7 (6.8)
Follow-up care			
Visits at index hospital, n (%)	4,832,894(79)	11,841 (81)	3139 (73)
Visits at referral hospital, n (%)	N/A	10,502 (72)	3266 (76)
30-day Mortality, n (%)	82,259 (1.3)	422 (2.9)	65 (1.5)

*Abbreviations*: *y* years, *SD* standard deviation, *ED* emergency department, *d* days, *IQR* interquartile range

### Primary transfer diagnoses

**Fig. 2:**
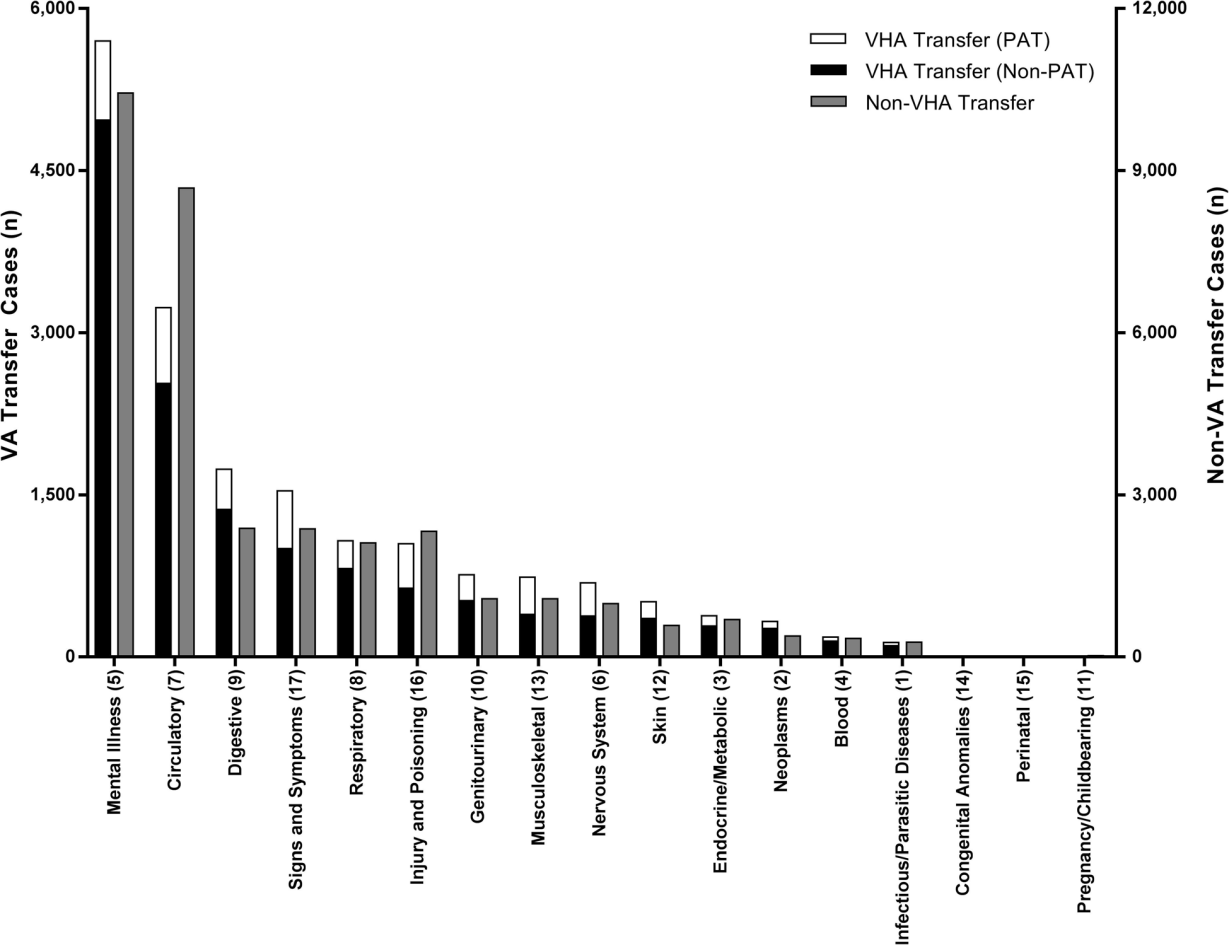
Distribution of inter-facility transfers by Clinical Classification Software (CCS) diagnosis group. Each bar shows the number of transfers within each diagnosis group. The left bar (black and white), shows the number of transfers to Veterans Health Administration (VHA) facilities, stratified by potentially avoidable transfer (PAT) status (left vertical axis). The right bar shows the number of non-VHA transfers (right vertical axis). The relative height of the black/white bar and the grey bar shows compares the distributions in transfers to VHA facilities vs. non-VHA facilities. Categories (horizontal axis) are CCS categories, with the CCS category number listed in parentheses after each category

The 3 diagnostic categories with most VHA transfers were mental health (CCS category 5, *n* = 6410 [34%]), cardiac (CCS category 7.2, *n* = 2161 [12%]), and digestive (CCS category 9, *n* = 1678 [9%]) conditions, with these top 3 categories comprising 55% of all transfers. The top ICD-9 diagnosis related to VHA ED transfer was suicidal ideation (V62.84). Among patients transferred for a procedure, interventional cardiac procedures were most common, constituting 45% of all procedures (Additional file 2: Table S1). The distribution of diagnoses for Veterans who were transferred to non-VHA hospitals is similar to those transferred to VHA facilities, with the exception of transfers for circulatory conditions, which were more prevalent among non-VHA transfers (Fig. 2).

### Primary diagnoses related to potentially avoidable transfer

Of all VHA transfers, the diagnostic categories associated with most PAT were mental health (*n* = 722 [11% potentially avoidable]) and cardiac (*n* = 452 [21% potentially avoidable]) diseases (Fig. 2).

### Health systems factors associated with potentially avoidable transfer

Transferred Veterans were more commonly transferred from smaller index VHA EDs (12.1 vs. 15.8 beds, *p* < 0.001), but smaller EDs did not have a higher prevalence of PAT (12.6 beds for PAT vs. 12.0 beds for non-PAT transfers, *p* < 0.001). That finding suggests that while smaller hospitals transfer a greater proportion of patients, ED size was not associated with transfer appropriateness. Hospitals staffed by at least 50% board-certified emergency physicians had a lower proportion of VHA transfers than those that did not (0.2% vs. 0.4%, *p* < 0.001), but the proportion of PAT was *higher* in hospitals with more than 50% board-certified emergency physicians (25% vs. 21%, *p* < 0.001). Patients were more likely to be transferred from hospitals that did not accept incoming unscheduled ambulance traffic (e.g., 911 calls) into the ED (47% vs. 27%, *p* < 0.001). For interpretation of computed tomography (CT) studies, transferring hospitals were more likely to use tele-radiology between 8 am and 5 pm Monday-Friday (8% vs. 4%) and were less likely to have radiology residents interpreting studies on nights and weekends (10% vs. 21%), which is a proxy for the staffing and availability of specialized services at these facilities.

Patients who were transferred to other VHA hospitals were likely to follow-up within 45 days at the referral hospital (73%), but they were equally likely to follow up at the index hospital compared with those who were not transferred (79% vs. 79%).

In summarizing data from the HAIG survey, 48% of medical directors of VHA EDs cited the transfer process as overly burdensome, with 29% identifying difficulties with identifying an accepting physician/facility, 13% identifying challenges with obtaining approval for transfer, and over 65% noting that administrative processes contribute to delay in transfer.

### Geographic factors

**Fig. 3:**
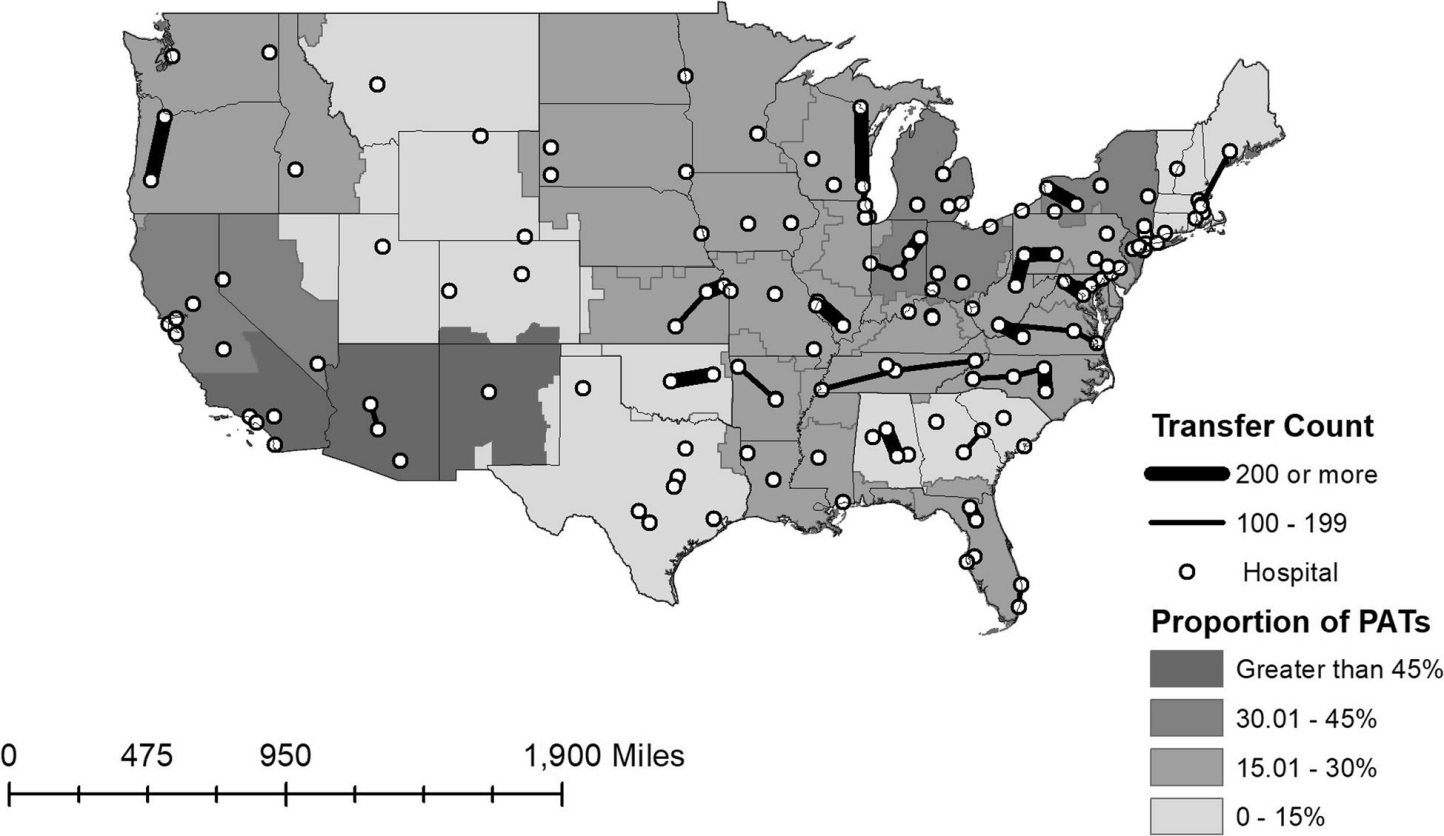
Map of ED-based VHA-to-VHA inter-facility transfers, 2012–2014. Each dot on the map indicates a single VHA hospital with an emergency department (ED). Lines between these hospitals indicate transfers between facilities, with the thickness of the line represents the number of transfers. For some pairs of hospitals, the number of transfers are bidirectional, in which case the number of transfers in each direction are added together to represent the total transfer volume. Lines are not drawn between hospitals that have fewer than 100 VHA-to-VHA transfers over the study period. The proportion of transfers within each Veterans Integrated Service Network (VISN) that qualify as potentially avoidable transfers (PAT) is represented by grayscale shading (see legend). Note that non-VHA transfers are not included on this figure. The authors would like to acknowledge Morgan Swanson, BS for her assistance with preparation of this figure

Variation in transfer proportions across index hospitals was high, ranging from 0 to 6% of total patients presenting to the ED who were transferred. PAT also varied widely, and PAT was not related to total transfer volume. Many high-transfer hospitals have an accompanying hospital that accepts the majority of transfers, but some sites have multiple transfer destinations (Fig. 3). There was also regional variation, with regional-level data showing transfer proportions that ranged from 8 to 53%.

**Fig. 4:**
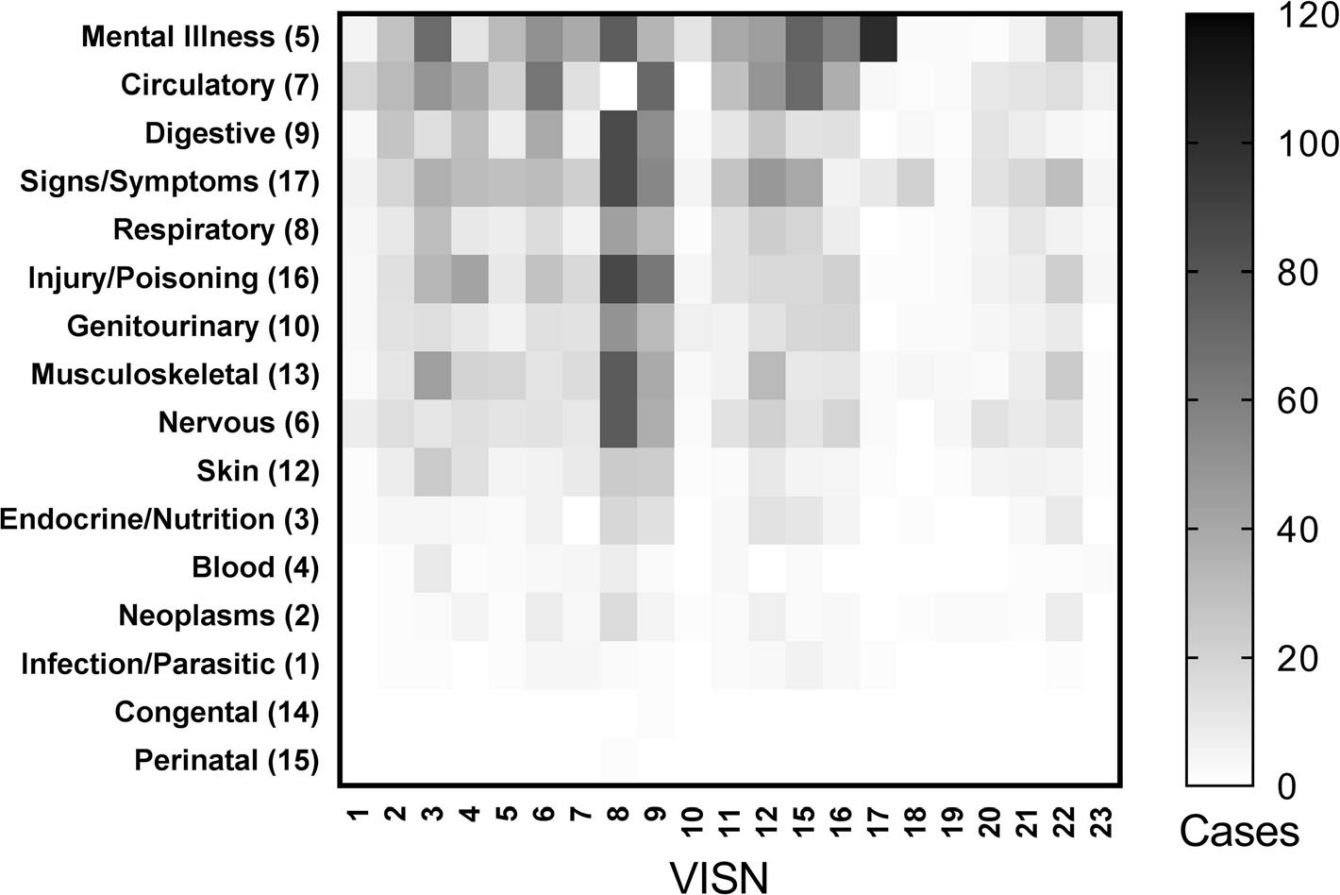
Regional variation in potentially avoidable transfers (PATs) by Clinical Classification Software (CCS) diagnosis group. Each cell in the heat map represents the total number of potentially avoidable transfers within one Veterans Integrated Service Network (VISN) region. CCS diagnosis categories are listed on the vertical axis, with the diagnosis group number listed in parentheses after the CCS category abbreviation. VISN regions are listed on the horizontal axis. Darker colors represent more PATs within the VISN for the diagnosis group

Although mental health-related transfers were the most prevalent condition for which VHA-to-VHA transfer was performed, in Veteran Integrated Service Network (VISN) numbers 7 (Alabama, Georgia, and South Carolina) and 17 (Texas), mental health transfers comprise over 60% of total inter-facility transfers. Similarly, circulatory system conditions comprise over 20% of total transfers VISN 1 (Connecticut, Maine, Massachusetts, Rhode Island, and Vermont), 6 (North Carolina and Virginia), and 15 (Kansas, Missouri, Southern Illinois and Indiana). Figure 4 shows VISN-level variation in transfer volume and diagnosis-specific transfers.

### Multivariable explanatory model

Adjusting for factors associated with inter-facility transfer, ED arrival at the index hospital on nights and weekends (aOR 1.252) and hospitals with more than 50% board-certified emergency physicians (aOR 1.266) both were associated with increased probability of PAT, while rural patients were at lower risk (aOR 0.798). The diagnostic categories associated with the highest adjusted risk were nervous system diseases (aOR 1.617), musculoskeletal conditions (aOR 1.571), and injury and poisoning (aOR 1.374). Importantly, however, the groups at highest risk did not constitute the greatest absolute number of PATs (Table 2).

**Table 2:** Multivariable explanatory model for potentially avoidable transfer (PAT)

Variable	Odds Ratio (95%CI)	*p*
Rural Residence	0.798 (0.715–0.890)	< 0.001
Arrival during Nights (5p-8a) or Weekend	1.252 (1.144–1.370)	< 0.001
Diagnosis Group (CCS)		< 0.001
Mental Illness (5)	0.238 (0.202–0.282)	
Circulatory (7)	0.518 (0.437–0.615)	
Digestive (9)	0.563 (0.464–0.683)	
Signs and Symptoms (17)	1.000 (ref)	
Respiratory (8)	0.568 (0.456–0.708)	
Injury and Poisoning (16)	1.374 (1.123–1.680)	
Genitourinary (10)	0.789 (0.619–1.004)	
Musculoskeletal (13)	1.571 (1.252–1.972)	
Nervous System (6)	1.617 (1.288–2.030)	
Skin (12)	0.870 (0.667–1.136)	
Endocrine/Metabolic (3)	0.535 (0.380–0.752)	
Neoplasms (2)	0.470 (0.321–0.690)	
Blood (4)	0.511 (0.327–0.798)	
Infectious/Parasitic Diseases (1)	0.380 (0.209–0.693)	
VISN		< 0.001
1	0.480 (0.276–0.834)	
2	1.280 (0.863–2.206)	
3	1.133 (0.712–1.804)	
4	0.534 (0.333–0.857)	
5	0.515 (0.319–0.832)	
6	0.706 (0.450–1.109)	
7	0.516 (0.320–0.834)	
8	0.839 (0.544–1.293)	
9	0.787 (0.505–1.227)	
10	1.791 (0.967–3.316)	
11	0.568 (0.349–0.923)	
12	0.613 (0.375–1.001)	
15	0.896 (0.553–1.454)	
16	0.492 (0.291–0.830)	
17	0.380 (0.236–0.611)	
18	0.788 (0.471–1.320)	
19	0.412 (0.215–0.790)	
20	0.459 (0.266–0.793)	
21	0.957 (0.565–1.622)	
22	1.673 (1.011–2.770)	
23	1.000 (ref)	
More than 50% Board-Certified Emergency Physicians	1.266 (1.103–1.452)	< 0.001

*Abbreviations*: *CCS* Clinical Classification Software, *VISN* Veterans Integrated Service Network

### Mental health subgroup analysis

Because of the high prevalence of mental health-related transfers, we conducted a post hoc subgroup analysis to identify factors associated with PAT within this group. The strongest risk factor for PAT (compared with both appropriate transfer and non-transfer) was being treated at night or on the weekend (67% vs. 63%, *p* < 0.001). Characteristics of the subset of patients transferred for mental health are summarized in Additional file 3: Table S2.

## Discussion

Our study identified several factors associated with inter-facility transfer, and mental health and cardiac disease are the two diagnosis groups for which inter-facility transfer is most prevalent in VHA hospitals. These findings highlight important differences between VHA healthcare and civilian healthcare systems, emphasizing the resources available within the VHA health system might be unique and underlining the need for VHA-specific solutions to health care delivery challenges. We also found that a sizeable portion of ED transfers from VHA hospitals refer patients outside the VHA.

The overall purpose of conducting this analysis was to identify areas where novel delivery of specialty care might avoid the need for some VHA transfers. In civilian health systems, ED-based telemedicine has been used to provide specialty provider and nursing support with the goal of improving transfer appropriateness [23–31]. This service has been most broadly implemented in tele-stroke care, providing real-time video consultation by a neurologist to an ED for the purpose of selecting patients for intravenous thrombolysis [32]. In some cases, inter-facility transfers have been avoided with targeted remote care [33–36] and allowed patients to remain near their families [37],which suggests that specialty telehealth capabilities may improve access, timeliness, and reduce the need for some emergency inter-facility transfers [23]. Some reports have even reported favorable provider-based outcomes related to telehealth implementation [38, 39].

Notably, over one-third of all VHA-to-VHA inter-facility transfers are for patients with mental health diagnoses, higher than that reported in civilian hospitals. This prevalence could be related to a combination of (1) robust mental health resources available within the VHA and (2) limited local bed availability or high inpatient occupancy necessitating transfer for inpatient hospitalization. Compared with civilian hospitals, VHA facilities have fewer patients transferred with myocardial infarction, stroke, and traumatic injury [11].

The overall goal of this project was to identify populations within the VHA where unnecessary transfers could be avoided. From our data, mental health diagnoses represent a rich target population for which telehealth might offer a plausible solution. Importantly, we feel that targeting mental health transfers is important because of the size of the population, despite the fact that the raw risk of PAT among that population is not elevated. Mental health providers are in critical shortage in most of the US [40, 41], and patients with mental health crises often require emergency care [42]. Increasingly, specialty mental health evaluation is unavailable, especially during evenings and weekends [43, 44]. Real-time telemedicine has been used for psychiatric consultations, counseling, and ongoing care as a strategy to leverage limited psychiatric resources across geographic areas, and these networks have even provided care in emergency departments [40, 45–51]. Providing detailed psychiatric evaluation, treatment recommendations, and disposition guidance could be one important service that could improve cost-effective access to mental health professionals, especially for rural veterans. Emergency mental health care may be particularly amenable to telehealth interventions, whereas cardiac catheterization and gastrointestinal endoscopy may not.

While myocardial infarction, stroke, and digestive conditions represented many transfers and many could be potentially avoidable, these conditions also commonly require procedural capabilities, and the need for those procedures may not be immediately obvious. Future work could develop additional interventions to better target inter-facility transfer in the group of patients needing cardiac catheterization, stroke care, or endoscopy, but these populations remain relatively small. Future work could also partner with local EMS services to better guide prehospital diversion practices. Despite the enriched mental health transfer population within VHA facilities, patients with mental health emergencies may benefit from provider-to-provider telemedicine in civilian hospitals as well, because factors driving inter-facility transfer and barriers to mental health access are ubiquitous outside the VHA.

A final important observation from this study surrounds the results from the HAIG survey of ED medical directors. Nearly half of medical directors of VHA EDs cited the transfer process as overly burdensome, and the majority noted that administrative processes resulted in transfer delays. While inter-facility transfer is complex, efforts to reduce the administrative burdens could be one effective way for patients to reach definitive care more quickly and reduce boarding in VHA EDs. In this study, we did not examine timeliness of transfer or the prevalence or impact transfer boarding has on VHA EDs, but the volume of transfers suggests that future work should examine the impact of these relationships in more detail.

Our study has several limitations. While administrative data provides a large sample, it limits the information available for individual patients to those coded in administrative claims. The second limitation is our definition of a transfer. The advantage of using the linkage method of identifying transfers is that we have high confidence that patients actually ended up receiving care in the receiving hospital. There could be some patients, however, who were discharged from the ED and presented to a different ED that we have inappropriately classified as transfer. Third, many patients were transferred from VHA EDs to non-VHA hospitals. While we were unable to assess PAT in non-VHA transfers, this remains an important population for future study. It also may be a population for which the motivations and outcome of interhospital transfer may differ from VHA transfers. Finally, our narrowly defined definition of PAT is not equivalent to avoidable transfer and some PAT may have still been necessary. Inter-facility transfer is a complex decision that would require more detailed qualitative patient-level analysis to better characterize specific reasons driving clinical decision-making.

## Conclusions

In conclusion, inter-facility transfer occurs in 0.8% of VHA ED visits, and only one-quarter of these VHA-to-VHA transfers are potentially avoidable. Rural Veterans are at high risk of VHA-to-VHA inter-facility transfer, but these transfers are no more likely to be PAT than for urban Veterans. Patients within VHA EDs are commonly transferred for mental illness, cardiac diseases, and digestive diseases, and there is wide variability between regions and hospitals in their transfer practices. Future work will focus on better understanding the reasons for transfers and factors that influence transfer decision-making, and future interventions will seek to improve the appropriateness, communication, and administrative factors surrounding inter-facility transfer.

## Supplementary information

**Additional file 1.** Data dictionary for variables included in analysis.

**Additional file 2: Table S1.** Most common procedures performed after VHA-to-VHA inter-facility ED transfer.

**Additional file 3: Table S2.** Patient and hospital-level factors associated with VHA-to-VHA ED inter-facility transfer for mental health patients, 2012–2014.

## Data Availability

The data that support the findings of this study are available from the Veterans Health Administration, but restrictions apply to the availability of these data, which were used under license for the current study and so are not publicly available. Data are, however, available from the authors upon reasonable request and with permission of the Veterans Health Administration.

## Abbreviations

AHRQ: Agency for Healthcare Research and Quality
CCS: Clinical Classification Software
CDW: Clinical data warehouse
CPT: Current Procedural Terminology
CT: Computed tomography
ED: Emergency department
HAIG: Healthcare Analysis and Information Group
HCUP: Health Care Utilization Project
IQR: Inter-quartile range
PAT: Potentially avoidable transfer
STROBE: STrengthening the Reporting of OBservational Studies in Epidemiology
VHA: Veterans’ Health Administration
VISN: Veteran Integrated Service Network
