# Supplementary material for: Potentially avoidable inter-facilit transfer from Veterans Health Administration emergency departments: A cohort study
Source: BMC Health Serv Res. 2020 Feb 12;20:110. doi: 10.1186/s12913-020-4956-6 (PMC7014752; doi:10.1186/s12913-020-4956-6)
Supplement: Supplementary file 1 — Additional file 1. Data dictionary for variables included in analysis. [file 12913_2020_4956_MOESM1_ESM.docx]

**Additional file 1. Data dictionary for variables included in analysis.**

| Unique identifier  Age  Sex  Race  VISN identifier (index hospital)  Date and time of visit (day of week and time of day)  Date and time of ED disposition  Facility identifier (index hospital)  Facility identifier (receiving hospital)  Disposition (local inpatient, local observation, ED-to-ED transfer, ED-to-inpatient transfer, ED-to-non-VHA transfer, discharge, died)  Hospital Length-of-Stay  Patient rurality category  Diagnoses  Clinical Classification Software (primary diagnosis)  Procedures  Surgical Procedures (Surgery Flag Software)  Death within 30 days  Total miles for transfer  Number of beds in index ED  ED staffed by at least 50% board-certified emergency physicians  ED accepts 911 response  Off-hours teleradiology (for CT interpretation)  Off-hours radiology resident coverage (for CT interpretation)  Outpatient follow-up within 45 days at index hospital  Outpatient follow-up within 45 days at referral hospital |
| --- |

*Abbreviations: VISN, Veteran Integrated Service Network; ED, emergency department; VHA, Veteran’s Health Administration; CT, computed tomography*
