# Supplementary material for: Potentially avoidable inter-facilit transfer from Veterans Health Administration emergency departments: A cohort study
Source: BMC Health Serv Res. 2020 Feb 12;20:110. doi: 10.1186/s12913-020-4956-6 (PMC7014752; doi:10.1186/s12913-020-4956-6)
Supplement: Supplementary file 2 — Additional file 2: Table S1. Most common procedures performed after VHA-to-VHA inter-facility ED transfer. [file 12913_2020_4956_MOESM2_ESM.docx]

**Table S1. Most common procedures performed after VHA-to-VHA inter-facility ED transfer.**

| **Procedure (ICD-9 or CPT code)** | **N (%)** |
| --- | --- |
| PTCA (Percutaneous transluminal coronary angioplasty [PTCA]) (00.66) | 364 (19) |
| Insertion of drug-eluting coronary artery stent(s) (36.07) | 295 (15) |
| Single internal mammary-coronary artery bypass (36.15) | 98 (4) |
| Ureteral catheterization (59.8) | 84 (3) |
| Excisional debridement of wound, infection, or burn (86.22) | 83 (3) |
| Endoscopic sphincterotomy and papillotomy (51.85) | 80 (4) |
| Initial insertion of dual-chamber device (37.83) | 79 (4) |
| Laparoscopic cholecystectomy (51.23) | 73 (3) |
| Insertion of non-drug-eluting coronary artery stent(s) (36.06) | 67 (3) |
| Endoscopic polypectomy of large intestine (45.42) | 64 (3) |
