# Supplementary material for: Potentially avoidable inter-facilit transfer from Veterans Health Administration emergency departments: A cohort study
Source: BMC Health Serv Res. 2020 Feb 12;20:110. doi: 10.1186/s12913-020-4956-6 (PMC7014752; doi:10.1186/s12913-020-4956-6)
Supplement: Supplementary file 3 — Additional file 3: Table S2. Patient and hospital-level factors associated with VHA-to-VHA ED inter-facility transfer for mental health patients, 2012–2014. [file 12913_2020_4956_MOESM3_ESM.docx]

**Table S2. Patient and hospital-level factors associated with VHA-to-VHA ED inter-facility transfer for mental health patients, 2012-2014**

|  | Non-Transfer  (n=410,233) | Non-Avoidable Transfer (n=5,984) | Potentially Avoidable Transfer  (n=747) |
| --- | --- | --- | --- |
| Age, y (SD) | 51.7 (15.1) | 49.6 (13.3) | 47.8 (14.6) |
| Male, n (%) | 372,037 (91) | 5,420 (91) | 655 (88) |
| Rurality of Residence |  |  |  |
| Urban, n (%) | 349,201 (90) | 4,525 (85) | 579 (87) |
| Large Rural, n (%) | 21,147 (5) | 469 (9) | 42 (6) |
| Small Rural, n (%) | 9,623 (2) | 212 (4) | 26 (4) |
| Isolated Rural, n (%) | 6,545 (2) | 122 (2) | 16 (2) |
| Day of the Week |  |  |  |
| Monday, n (%) | 66,170 (16) | 963 (16) | 126 (17) |
| Tuesday, n (%) | 66,553 (16) | 920 (15) | 126 (17) |
| Wednesday, n (%) | 64,446 (16) | 882 (15) | 125 (17) |
| Thursday, n (%) | 62,827 (15) | 867 (14) | 112 (15) |
| Friday, n (%) | 63,726 (16) | 991 (15) | 61 (8) |
| Saturday, n (%) | 45,448 (11) | 714 (12) | 83 (11) |
| Sunday, n (%) | 41,063 (10) | 757 (13) | 114 (15) |
| Time of Day |  |  |  |
| 8a-5p Mon-Fri, n (%) | 156,038 (38) | 2,191 (37) | 249 (33) |
| Nights and Weekends, n (%) | 254,195 (62) | 3,793 (63) | 498 (67) |
| Transfer Location |  |  |  |
| ED, n (%) | N/A | 2,593 (43) | 530 (71) |
| Inpatient, n (%) | N/A | 3,391 (57) | 217 (29) |
| Hospitalization, n (%) | 187,248 (46) | 5,984 (100) | 321 (43) |
| Hospital Length of Stay, d (median, IQR) | 5 (6) | 6 (6) | 1 (0) |
| Number of ED beds | 16 (10) | 14 (14) | 14 (14) |
| Follow-up care |  |  |  |
| Visits at index hospital, n (%) | 351,847 (86) | 4,816 (80) | 559 (75) |
| Visits at referral hospital, n (%) | N/A | 4,558 (76) | 589 (79) |
| 30-day Mortality, n (%) | 2,799 (0.7) | 21 (0.4) | 1 (0.1) |
